# Autophagy as a potential mechanism underlying the biological effect of 1,25-Dihydroxyvitamin D3 on periodontitis: a narrative review

**DOI:** 10.1186/s12903-023-02802-9

**Published:** 2023-02-13

**Authors:** Xiaoting Chen, Zulema Arias, Kazuhiro Omori, Tadashi Yamamoto, Yuki Shinoda-Ito, Shogo Takashiba

**Affiliations:** 1 Department of Pathophysiology-Periodontal Science, Graduate School of Medicine, Dentistry and Pharmaceutical Sciences, Okayama University, 2-5-1 Shikata-Cho, Kita-Ku, Okayama, Japan; 2 Department of Periodontics and Endodontics, Okayama University Hospital, Okayama, Japan

**Keywords:** Vitamin D, Autophagy, Periodontitis, Epithelial barrier, Immunity, Inflammation, Alveolar bone loss

## Abstract

The major active form of vitamin D, 1,25-dihydroxyvitamin D3 (1,25D3), is known for its wide bioactivity in periodontal tissues. Although the exact mechanisms underlying its protective action against periodontitis remain unclear, recent studies have shown that 1,25D3 regulates autophagy. Autophagy is vital for intracellular pathogen invasion control, inflammation regulation, and bone metabolic balance in periodontal tissue homeostasis, and its regulation could be an interesting pathway for future periodontal studies. Since vitamin D deficiency is a worldwide health problem, its role as a potential regulator of autophagy provides new insights into periodontal diseases. Based on this premise, this narrative literature review aimed to investigate the possible connection between 1,25D3 and autophagy in periodontitis. A comprehensive literature search was conducted on PubMed using the following keywords (e.g., vitamin D, autophagy, periodontitis, pathogens, epithelial cells, immunity, inflammation, and bone loss). In this review, the latest studies on the protective action of 1,25D3 against periodontitis and the regulation of autophagy by 1,25D3 are summarized, and the potential role of 1,25D3-activated autophagy in the pathogenesis of periodontitis is analyzed. 1,25D3 can exert a protective effect against periodontitis through different signaling pathways in the pathogenesis of periodontitis, and at least part of this regulatory effect is achieved through the activation of the autophagic response. This review will help clarify the relationship between 1,25D3 and autophagy in the homeostasis of periodontal tissues and provide perspectives for researchers to optimize prevention and treatment strategies in the future.

## Background

Periodontitis is a complex infectious disease that destroys periodontal tissues and has various etiological and contributing factors. It is highly prevalent in populations worldwide [1]. The dynamic interaction between complex periodontal polymicrobial infection and destructive immune response is a pivotal pathogenic factor of periodontitis [1, 2]. Further improvements in the diagnosis and treatment of periodontitis are still needed, and new types of therapies with low cost and high impact should be developed [1]. There are over one billion people worldwide suffering from vitamin D (VD) deficiency, which is a global public health issue that cannot be underestimated [3]. Serum 25-hydroxyvitamin D [25(OH)D] level below 20 ng/mL (50 nmol/L) is defined as deficiency and 21–29 ng/mL (52.5–72.5 nmol/L) is insufficiency [4]. VD deficiency is related to the risk of periodontitis [5, 6]. Therefore, studying the role of VD in periodontal health is of great importance.

**Fig. 1:**
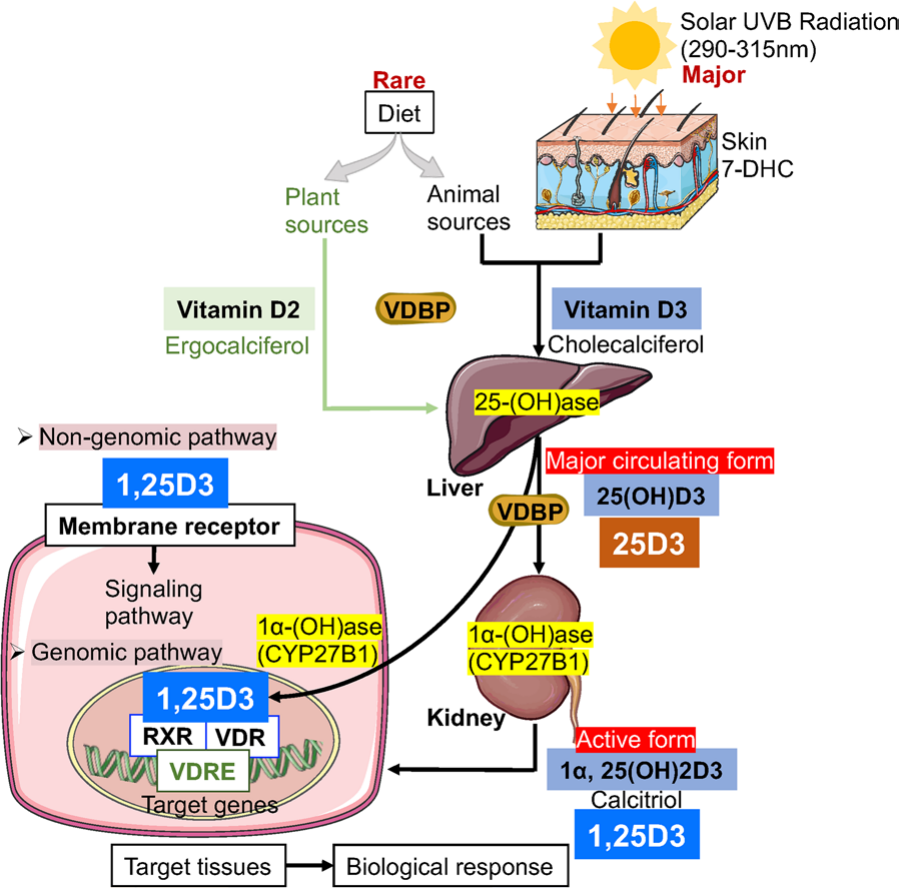
Metabolism of vitamin D and biological response with genomic and non-genomic effects. VD is formed mainly through exposure to solar ultraviolet B (UVB) radiation by 7-dehydrocholesterol (7-DHC) in the human skin and can also be derived from the diet. The amount of VD obtained from diets and supplements is very low. VD is delivered in circulation in combination with VD-binding proteins (VDBPs) to the liver, where it is converted to 25(OH)D by the action of vitamin D-25-hydroxylase (25-OHase). After the binding of 25(OH)D to VDBPs, it subsequently reaches the kidney or other tissues (such as epithelial cells) [7], where it is converted to the active form 1,25(OH)2D by 25-hydroxyvitamin D-1 hydroxylase (1-Ohase, CYP27B1). The most biologically active metabolite of VD is 1,25(OH)2D3 (1,25D3), which is derived from vitamin D3 (cholecalciferol) and exerts its biological effects mainly by binding to the VDR. In the nucleus, 1,25D3 can bind successively to the nuclear receptor VDR, retinoid X receptor (RXR), and VD response elements (VDREs), which affect the transcription of target genes, ultimately affecting protein synthesis and decomposition. In addition, 1,25D3 can bind to the membrane receptor membrane-associated, rapid response steroid (MARRS)-binding protein to exert a non-genetic effect by interacting with other signaling pathways

VD is a fat-soluble vitamin and precursor of steroid hormones. After two hydroxylations mainly in the liver and kidney (or some other tissues) [7], it is converted to its major active form, 1,25-dihydroxyvitamin D3 (1,25D3), which regulates a wide range of biological processes in target tissues through genomic and non-genomic pathways [8] (Fig. 1). Interestingly, the local vitamin D3 conversion to both 25(OH)D3 and 1,25(OH)2D3 in oral keratinocytes, human gingival fibroblasts (HGFs) and periodontal ligament cells (HPDLCs) was observed [9, 10]. Topical administration of inactive vitamin D3 showed a similar anti-inflammatory effect as 1,25(OH)2D3 did, indicating the possibility of the direct application of inactive vitamin D3 to the gingiva [10]. Furthermore, the biological functions of 1,25D3 are mainly achieved by binding to the vitamin D receptor (VDR), a member of the nuclear receptor superfamily that mediates the transcription of target genes (Fig. 1). It has been shown that VDR not only exists in classical small intestinal epithelial cells, bone cells, and kidney cells but also in various immune cells, tumor cells, and epithelial cells, revealing the pivotal role of 1,25D3 in many extra-skeletal diseases [8, 11]. Recently, 1,25D3 has become a hot topic in periodontitis research. Its important role in defending against microbial infection and modulating immune responses in the oral environment has been actively discussed. However, the exact underlying molecular mechanism remains unclear.

Autophagy, a highly conserved lysosomal degradation process, is central to maintaining organismal homeostasis. Alterations in autophagy have been associated with various diseases, including periodontitis [12]. Its potential role in the pathogenesis of periodontitis has been reported [13]. Hence, keeping autophagy homeostasis is important. In recent years, the development of autophagy modulators has attracted widespread interest; these modulators have shown great therapeutic potential for some related diseases [14]. Increasing evidence indicates that 1,25D3 can promote autophagy to protect against the development of infectious and inflammatory diseases [15]. In addition, experimental studies on the association between 1,25D3 and autophagy in periodontitis are still in their infancy and no comprehensive review has been published to analyze the possibility that autophagy regulation is involved in the 1,25D3-mediated protection against periodontitis. In view of the significance of the interplay between 1,25D3 and autophagy, this review summarizes the latest evidence related to (1) the protective mechanism of 1,25D3 against periodontitis discovered so far, (2) the connection between 1,25D3 and autophagy, and (3) possible roles of 1,25D3-modulated autophagy in the killing of pathogens, modulation of immune and inflammatory responses, and reduction of bone loss.

## Protective action of 1,25D3 against periodontitis

### Pathogen killing

**Fig. 2:**
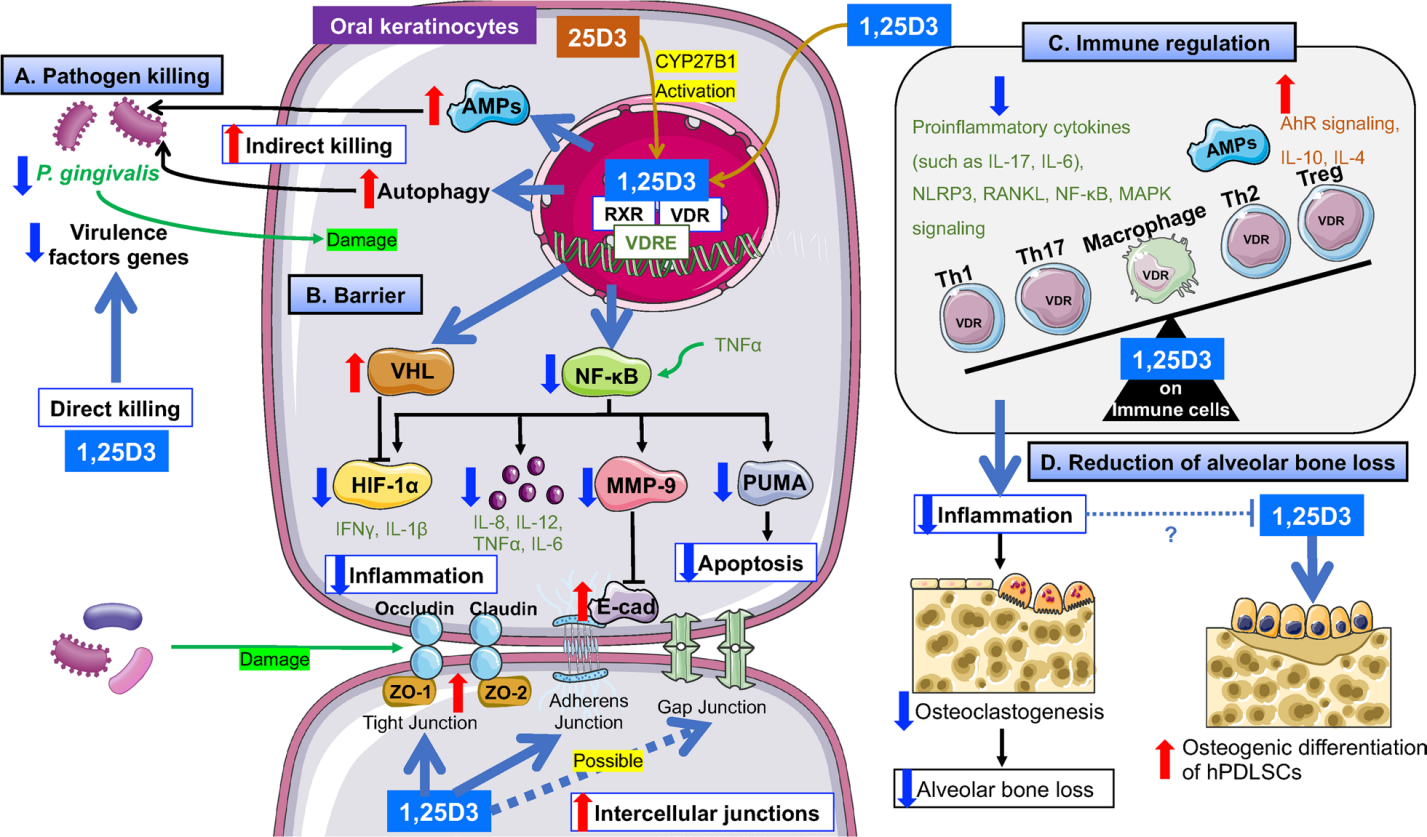
Possible mechanism by which 1,25D3 exerts biological effects on periodontal tissues. **A** 1,25D3 had a direct antimicrobial effect against specific pathogens by its lytic activity and inhibition of *P. gingivalis* virulence factors, and it also increased the expression levels of LL-37 and sIgA in the saliva. After *P. gingivalis* invasion, 1,25D3 induces functional autophagy to degrade *P. gingivalis,* upregulates AMPs gene expression to kill pathogens, exerting indirect antimicrobial action. **B** 1,25D3 impedes TNF-α-NF-κB signaling and upregulates VHL signaling to protect the epithelial barrier from pathogen invasion into deep tissues. Its protective role includes strengthened intercellular junctions, decreased inflammatory response (reduced levels of TNFα, IL-6, IL-12, IFNγ, IL-1β, and HIF-1α), and reduced keratinocyte apoptosis. In addition, 25(OH)D3 is converted to active 1,25D3 in gingival epithelial cells and subsequently exhibits its biological effects by binding to VDR. **C** 1,25D3 may exert its anti-inflammatory properties against *P. gingivalis* infection by regulating different signaling pathways in the macrophages/monocytes (such as NF-κB and MAPK) and increasing the polarization of Th cells to the Th2/Treg phenotype, accompanied by downregulation of some pro-inflammatory cytokines (such as IL-17 and IL-6) and upregulation of AMPs, AhR, IL-4, and IL-10. **D** 1,25D3 may exert its effect on alveolar bone via immune regulation, inhibition of osteoclastogenesis, induction of osteogenic differentiation, and transcriptional regulation of osteogenesis-related factors. However, its response to bone loss, such as the regulation of osteogenesis-related factors, may be locally diminished by inflammatory stimuli

Compared to antibiotics that may lead to bacterial resistance and some allergic reactions, 1,25D3 has a high safety profile because it modulates innate immunity (including antimicrobial peptides (AMPs) and autophagy) to exert antimicrobial effects and may also directly act on bacteria (Fig. 2A).

AMPs, including cathelicidin, β-defensins, and S100 proteins, are mainly produced by immune and epithelial cells [16]. LL-37, the only human member of the cathelicidin family, has antibacterial activity against various oral pathogens [17]. The cathelicidin antimicrobial peptide (CAMP) gene is a direct target of VDR-mediated transcription [18]. The antibacterial properties of 1,25D3 against *Aggregatibacter actinomycetemcomitans* (*A. actinomycetemcomitans*) might also result from 1,25D3-induced LL-37 [19]. When the concentration of the vitamin D biomarker 25(OH)D3 in serum was lower than 30 ng/mL in patients with dental caries, the levels of secretory immunoglobulin A (sIgA), LPS binding protein (LBP), cathelicidin and total antioxidant activity decreased. After VD supplementation (VDS), the levels returned to normal [20], and saliva LL-37 levels was related to serum concentration of vitamin D in six-year-old children [21]. These results demonstrate the important role of 1,25D3-induced AMPs, suggesting a potential association between VD deficiency and susceptibility to microbial infections.

Recently, a few studies have reported a direct effect of 1,25D3 on some bacterial cells. Due to its strong lipid solubility, cell membrane integrity might be altered, and permeability to other substances, such as antibiotics, might be increased [22, 23]. 1,25D3 exerts an inhibitory effect on *Fusobacterium nucleatum* (*F. nucleatum*), *A. actinomycetemcomitans*, *Solobacterium moorei*, and *Streptococcus mutans* (*S. mutans*) at high concentrations (≥ 100 μg/mL), whereas 1,25D3 has been found to exert specific antibacterial activity against *Porphyromonas gingivalis (P. gingivali*s) at very low concentrations (minimum inhibitory concentration [MIC]: 3.125 to 6.25 μg/mL, MBC: 6.25 to 25 μg/mL). In addition, 1,25D3 can significantly reduce the gene expression of virulence factors involved in bacterial colonization (fimA, hagA, and hagB) and factors involved in tissue destruction (rgpA, rgpB, and kgp) [24]. Unlike antibiotics that target the in vitro viability of bacteria, targeting bacterial virulence factor genes that are critical for in vivo viability can reduce bacterial resistance–another valuable alternative antibacterial approach. Interestingly, 1,25D3 was found to exert a partial synergistic effect against *P. gingivalis* when combined with metronidazole. In combination with tetracycline, 1,25D3 showed an additive effect [24].

### Epithelial barrier

The oral epithelial barrier separates the host from the oral environment, and the body’s natural physiological barrier prevents pathogens and exogenous substances from entering the deep tissues. In the gingival epithelium, oral keratinocytes are the primary cell type connected by various transmembrane proteins with special structures and functions, such as tight junctions, adherens junctions, and gap junctions [25]. Tight junctions are semi-permeable barriers composed of claudin, occludin, and zonula occludens (ZO)-1-3. Adherens junctions consist of transmembrane cadherin (mainly E-cadherin) and intracellular catenin [25]. VDR is expressed in the entire gingival epithelial layer [26]. 1,25D3/VDR signaling regulates the expression of different proteins involved in intercellular junctions (including claudin, occludin, ZO-1/2, E-cadherin, and β-catenin) to maintain epithelial barrier integrity [27, 28]. In human oral keratinocytes, E-cadherin intercellular junctions (ECJs) can be dissociated by matrix metalloproteinase 9 (MMP-9) induced by tumor necrosis factor-α (TNF-α) [26]. Furthermore, 1,25D3 can reduce MMP-9 production by inhibiting nuclear factor-κB (NF-κB) signaling, thus attenuating the downregulation of ECJs and enhancing intercellular junctions [26].

Increased apoptosis of oral epithelial cells can disrupt the mucosal barrier and accelerate bacterial invasion. 1,25D3/VDR reduces oral keratinocyte apoptosis by inhibiting the activation of NF-κB-dependent p53-upregulated modulator of apoptosis (PUMA), which is a key pro-apoptotic regulator. In addition, other apoptogenic factors induced by *Escherichia coli* LPS, including phospho-p65 (p-p65) and active caspase 3/9, were also reduced by 1,25D3 [29].

1,25D3/VDR can reduce the inflammatory response in both von Hippel-Lindau (VHL)- and NF-κB-dependent pathways [29, 30]. Human oral keratinocytes stimulated by LPS can produce a large amount of hypoxia-inducible factor-1α (HIF-1α), and four key cytokines (interferon-γ [IFNγ], interleukin-1β [IL-1β], TNFα, and IL-6) [30]. HIF-1α increases cytokine transcription and accelerates inflammatory responses [31]. The overexpression of HIF-1α and inflammatory cytokines in human oral keratinocytes were found to be reversed by 1,25D3 treatment via impeded NF-κB signaling pathway and upregulated VHL expression; however, whether 1,25D3 has a direct regulatory effect on HIF-1α remains unknown. IFNγ and IL-1β expression can be reduced by 1,25D3 in a HIF-1α-dependent pathway, while the downregulation of TNF-α and IL-6 may occur through the inhibition of the NF-κB signaling pathway [30]. An in vivo study found that the lack of 1,25D3 in oral epithelial cells exacerbates the inflammatory response induced by LPS in the gingival epithelium. Furthermore, IL-1α mRNA levels were inhibited in oral keratinocytes treated with 10 nM of 1,25D3 [10]. The expression of other pro-inflammatory cytokines IL-8 and IL-12 was decreased by 1,25D3 treatment in human gingival epithelial cells (HGECs) infected with *P. gingivalis*, while in other periodontal tissue cells, such as HGFs and HPDLCs, 1,25D3 was also found to reduce inflammatory levels [32, 33].

These findings suggest that the inhibition of NF-κB signaling by 1,25D3 plays an important role in enhancing intercellular junctions, reducing apoptosis, and relieving the inflammatory response in oral epithelial cells (Fig. 2B).

### Immune and inflammation regulation

The development of periodontitis caused by oral pathogen infection is related to the inflammatory mediators locally produced during the host immune process. Regarding VDS, it is recommended that patients with VD deficiency should be administered VDS before periodontal surgery to avoid the negative effects on the treatment outcomes [34]. Recently, in some in vivo studies, VDS significantly reduced inflammatory response and alveolar bone loss [10, 35, 36]. However, the modest effect of 1,25D3 on periodontitis with limited clinical relevance was also reported [37], which may partly result from different standardized criteria, study populations, short follow-up period and study design. Thus, the long-term efficacy and standardized criteria of VDS as an adjuvant therapy for periodontal treatment need to be further studied. In addition to HGECs, HGFs and HPDLCs mentioned above, 1,25D3/VDR signaling in various immune cells also participates in the defense mechanism against pathogens invasion and inflammatory response (Fig. 2C).

In the case of innate immunity, 1,25D3 regulates different signaling pathways and cytokine expressions in monocytes/macrophages to exert specific anti-inflammatory properties against *P. gingivalis* infection. 1,25D3 inhibits the activation of NF-κB [24], p38 mitogen-activated protein kinase (MAPK), and extracellular signal-regulated kinase-1/2 (ERK-1/2) signaling pathway [38]. 1,25D3 can also inhibit the expression of IL-6 while elevating the expression of IL-10 [38, 39]. In addition, studies have found that in patients with type 2 diabetes mellitus and periodontitis, 1,25D3 may promote neutrophil apoptosis through the p38-MAPK pathway [40]. In addition, in the case of adaptive immunity, 1,25D3 regulates the differentiation of T lymphocytes, secretion of immunoglobulin, and production of inflammatory cytokines [41]. 1,25D3 intervention can regulate Th cell polarization toward different subsets. Polarized subsets, especially Th1, Th17, Th2, and Treg subsets, together with secreted cytokines, are key players in the destructive and reparative phases of periodontitis [42]. 1,25D3 decreased the proportions of Th1 and Th17 cells, increased the proportions of Th2 and Treg subsets, downregulated IL-17 levels, and upregulated IL-4 and IL-10 levels [42, 43].

### Reduction of alveolar bone loss

1,25D3 may exert its effects on alveolar bone via its immunomodulatory effect, inhibition of osteoclastogenesis, induction of osteogenic differentiation, and transcriptional regulation of osteogenesis-related factors (Fig. 2D). One study reported increased alveolar bone resorption with a VD intake of less than 400 IU/d and a reduced risk of severe chronic periodontitis with a VD intake of more than 800 IU/d [44]. In in vivo experiments, the addition of 1,25D3 reduced bone loss, possibly because of the inhibition of the inflammatory response [36, 45, 46]. In gingival epithelium, after 1,25D3 addition, the expression of VDR and aryl hydrocarbon receptor (AhR) signaling was upregulated, and subsequently, LPS-induced activation of NF-κB and the nucleotide-binding oligomerization domain-like receptor family pyrin domain containing 3 (NLRP3) inflammasome were suppressed [36]. AhR is widely expressed in immune cells and has been identified as a potential target for immunomodulation [47]. NLRP3 inflammasome is closely associated with periodontal damage. In macrophages, activated AhR signaling blocks the activation of the NLRP3 inflammasome by NF-κB, and subsequent production of inflammatory cytokines is inhibited [48]. The expressions of IL-1β and IL-6 were downregulated, possibly due to the regulation of the inflammasome pathway by 1,25D3.

As mentioned previously, 1,25D3 administration inhibited alveolar bone resorption activity by modulating the polarization of Th cells in experimental periodontitis [43]. Further studies demonstrated the potential association between the effect of 1,25D3 on Th cells and osteoclast activation. In an inflammatory environment, the expressions of osteoclastogenesis-related markers (such as MMP-9) and RANKL in vitro are downregulated by 1,25D3 via the regulation of Th cell subsets [42]; thus, osteoclastogenesis is inhibited.

In addition, 1,25D3 has been shown to significantly promote osteogenic differentiation of human periodontal ligament stromal cells/stem cells (hPDLSCs) and increase the expression of osteogenesis-related factors (osteocalcin and osteopontin) [49, 50]. However, inflammatory stimulation was recently found to diminish the 1,25D3-induced expression of osteocalcin and osteopontin in hPDLSCs [49], which may be a result of the inhibited transcriptional activity of VDR [51]. This study had some limitations due to the addition of an artificial additive, such as dexamethasone, to the osteogenic induction medium, which may have influenced the results. Future in-depth research on the mechanisms by which the inflammatory responses affect the bioactivity of 1,25D3 may help improve the effectiveness of VDS as an adjunctive periodontal therapy.

## 1,25D3 and autophagy

### Autophagy

**Fig. 3:**
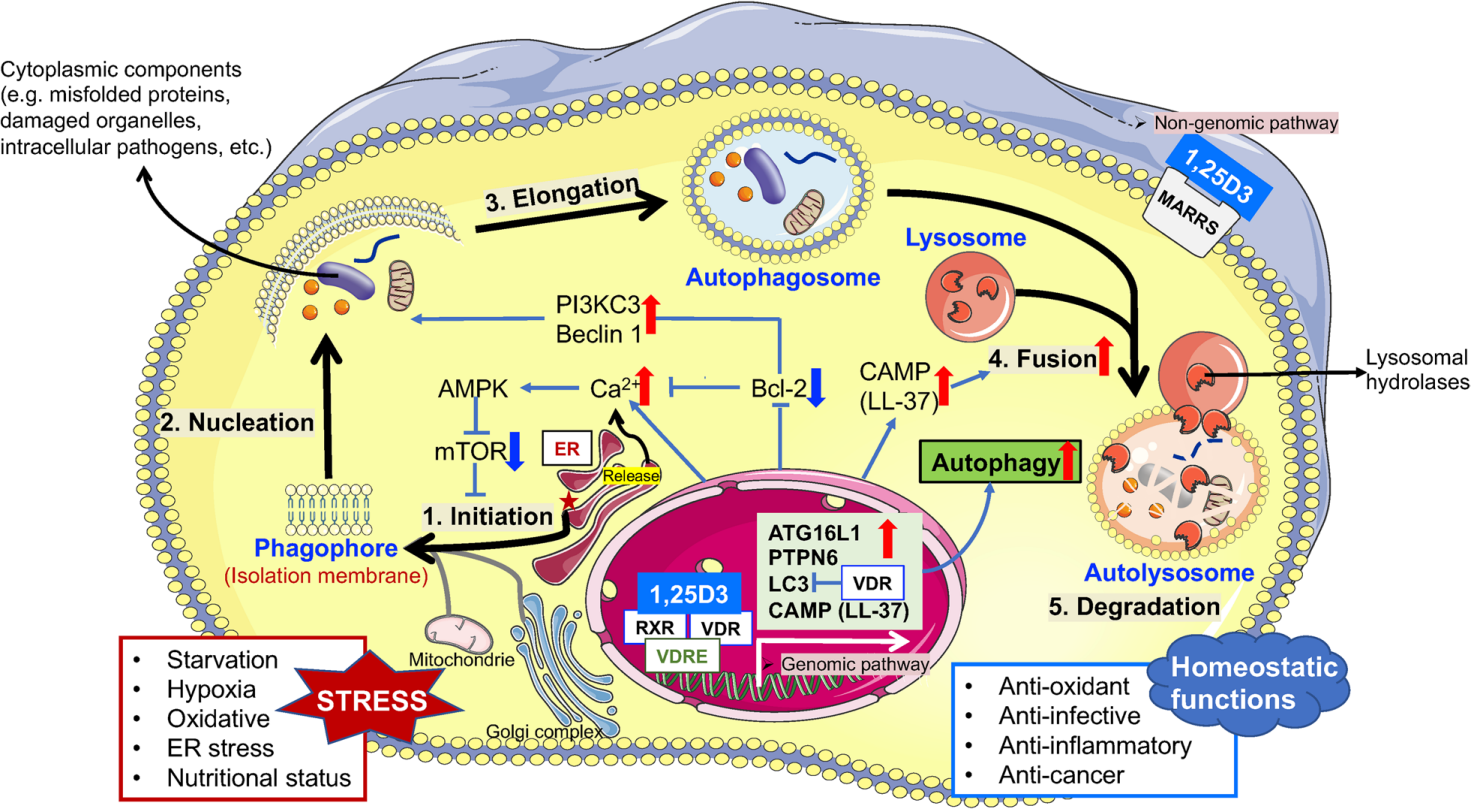
General regulatory mechanism of 1,25D3/VDR on autophagy. The classic macroautophagic process is induced by different stress signals and consists of five steps: (1) Phagophore (or isolation membrane) initiation from the endoplasmic reticulum (ER), and other different cellular membranes, including the Golgi complex, mitochondria, and plasma membrane may also deliver phospholipids to phagophore; (2) phagophore nucleation; (3) phagophore elongation forming an autophagosome after closure; (4) fusion of autophagosome and lysosome forming an autolysosome; and (5) degradation of cytoplasmic components within the autolysosome. Through genomic and non-genomic pathways, 1,25D3 induces autophagy at different steps. 1,25D3 increases cytosolic-free calcium that is released from ER and inhibited by Bcl-2, and it downregulates mTOR expression to initiate autophagy induction, regulates PI3KC3/Beclin-1 pathway to affect phagophore nucleation, and upregulates human cathelicidin (LL-37) to promote the fusion of the lysosome and autophagosome. Besides, 1,25D3 can transcriptionally upregulate the gene expressions of *ATG16L1*, *PTPN6*, *LC3,* and *CAMP* to induce autophagy. 1,25D3 de-represses the LC3B gene (*MAP1LC31B*) by VDR. These pathways found in different cell and tissue types induce autophagy and play a protective role in different diseases through antioxidant, anti-infective, anti-inflammatory, and anticancer effects

Autophagy is a major intracellular degradation process in which cytoplasmic components (misfolded proteins, internalized pathogens, and damaged organelles) are delivered to lysosomes for degradation [52]. Autophagy generates energy for cell renovation, maintains cellular homeostasis, and participates in various biological processes. In mammals, according to the different pathways in which cellular components are delivered to lysosomes, autophagy is mainly divided into three categories: macroautophagy, microautophagy, and chaperone-mediated autophagy. Since macroautophagy is the main way of regulating cellular physiological activity, in this review, we will simply refer to macroautophagy as “autophagy.” The autophagy process involves five major steps (Fig. 3): initiation, elongation, maturation, fusion with lysosomes, and degradation [53]. The isolated membrane structure that wraps the target contents gradually expands to form a unique double-layer membrane structure, namely the autophagosome. Subsequently, the lysosomes and autophagosomes fuse to form an autolysosome, which becomes a monolayer membrane structure, and the target content is degraded by lysosomal hydrolases to meet the needs of cell metabolism, renewal of these organelles, and removal of pathogens [52].

### Regulatory effect of 1,25D3 on autophagy

In recent years, many studies have found that in addition to affecting calcium and phosphorus metabolism and regulating immunity and infection, 1,25D3 also mediates autophagy via genomic and non-genomic signaling pathways to influence the physiological functions of different organs [15]. VD deficiency also affects autophagy [54]. However, its specific mechanism of action remains unclear. At present, related research has mainly focused on regulating cytosolic calcium levels, autophagy-related gene expression, AMPs, and lysosomes. 1,25D3-induced autophagy signaling has been reported to play a protective role in various diseases through its antioxidant, anti-infective, anti-inflammatory, and anticancer effects [15].

In detail, 1,25D3/VDR can induce autophagy by increasing the levels of cytosolic free calcium and downregulating the expression of mammalian target of rapamycin (mTOR) and Bcl-2 which represses Ca^2+^ release [15]. Regulation of the class III phosphoinositide 3-kinase (PI3KC3)/Beclin-1 pathway by 1,25D3 in different cells and tissues influences autophagosome nucleation [55, 56]. Beclin-1, a core component of the P13K complex involved in autophagosome nucleation and maturation, is a key regulator of autophagy and is affected by NF-κB, Bcl-2, 1,25D3, and 1,25D3 analogs [57]. Additionally, 1,25D3/VDR induces CAMP synthesis and activates autophagy in *Mycobacterium tuberculosis* (Mtb)-infected monocytes. Cathelicidin LL-37 is a downstream target gene that promotes the fusion of autophagosomes and lysosomes to form autolysosome [58]. Moreover, 1,25D3-induced human cathelicidin LL-37 was found to promote human monocyte autophagy via transcriptional activation of Beclin-1 and autophagy-related (ATG) 5 [58]. Another recent study revealed 1,25D3-VDR-PTPN6 axis-regulated autophagy in macrophages. Protein tyrosine phosphatase non-receptor type 6 (PTPN6), a cytoplasmic phosphatase, is induced by 1,25D3 and regulates autophagy-related genes to promote 1,25D3-mediated autophagy [59]. In addition, 1,25D3/VDR was found to promote the transcriptional upregulation of ATG16L1 to affect autophagy [60]. Treatment with 1,25D3 increases basal levels of autophagy by de-repressing the key autophagy gene LC3B (*MAP1LC31B*) which is constitutively repressed by VDR [61] (Fig. 3). Interestingly, 1,25D3 can also reduce autophagy by decreasing the levels of NF-κB, TNF-α, or IFN-γ [62], which indicates that regulation of autophagy by 1,25D3/VDR signaling is bidirectional and may vary in different infectious diseases.

It was found that compared to healthy gingival tissues, inflammatory sites from naturally occurring periodontitis of rhesus monkeys showed significant alterations in the expression of some autophagy-related genes, suggesting that autophagy may be impaired in periodontal lesions and involved in the pathogenesis of periodontitis [12]. Other human clinical studies have also found significant differences in the levels of autophagy between healthy periodontal subjects and patients with periodontitis. For example, peripheral blood mononuclear cells (PBMCs) from patients with periodontitis showed significantly downregulated levels of the autophagy-related proteins ATG5-12 conjugate, ATG16L1, and ATG7. The regulation of autophagy is therefore a potential therapeutic target for periodontitis in the future. A study showed that vitamin D supplementation enhanced autophagy by upregulating the expression of these proteins in PBMCs and upregulating the expression of ATG5 and ATG16L1 in gingival tissue from patients with periodontitis [35]. This study had the limitation of a small sample size and selected patients without initial VD deficiency. In addition, clinical studies have also found that inflammatory periodontal tissue and peripheral blood in patients with periodontitis showed a higher LC3 II/I ratio relative to healthy periodontium [63, 64]. An in vitro study found that vitamin D3 supplementation further increased the LC3 II/I ratio upregulated by Pg [65]. It has been mentioned that the general effect of vitamin D on autophagy is bidirectional. Therefore, more in vivo and in vitro experiments are needed to verify the association between vitamin D and autophagy in periodontitis in order to develop a new therapeutic strategy for periodontitis.

## Possible role of 1,25D3 via autophagy modulation in periodontitis

Although the specific mechanism remains unclear, there have been already some in vivo and in vitro studies supporting the hypothesis of the involvement of autophagy regulation in the protective effects of vitamin D in other infectious and inflammatory diseases such as *Salmonella* colitis [66], UV-mediated sunburn and inflammation [67], allergic airway inflammation [68], and osteoarthritis [69]. The potential role of 1,25D3-induced autophagy signaling in different cell and tissue types was discussed in a recent review [15]. However, little information is available about its role in oral health. Existing studies provide sufficient evidence to support the multidimensional regulatory role of autophagy in the pathogenesis of periodontitis, including the regulation of pathogen invasion, immunity, inflammation, and alveolar bone homeostasis. 1,25D3, a key regulator of autophagy, shows great potential in preventing and alleviating pathological responses in periodontitis, which is mediated, at least in part, via the modulation of autophagy.

### Barrier

**Fig. 4:**
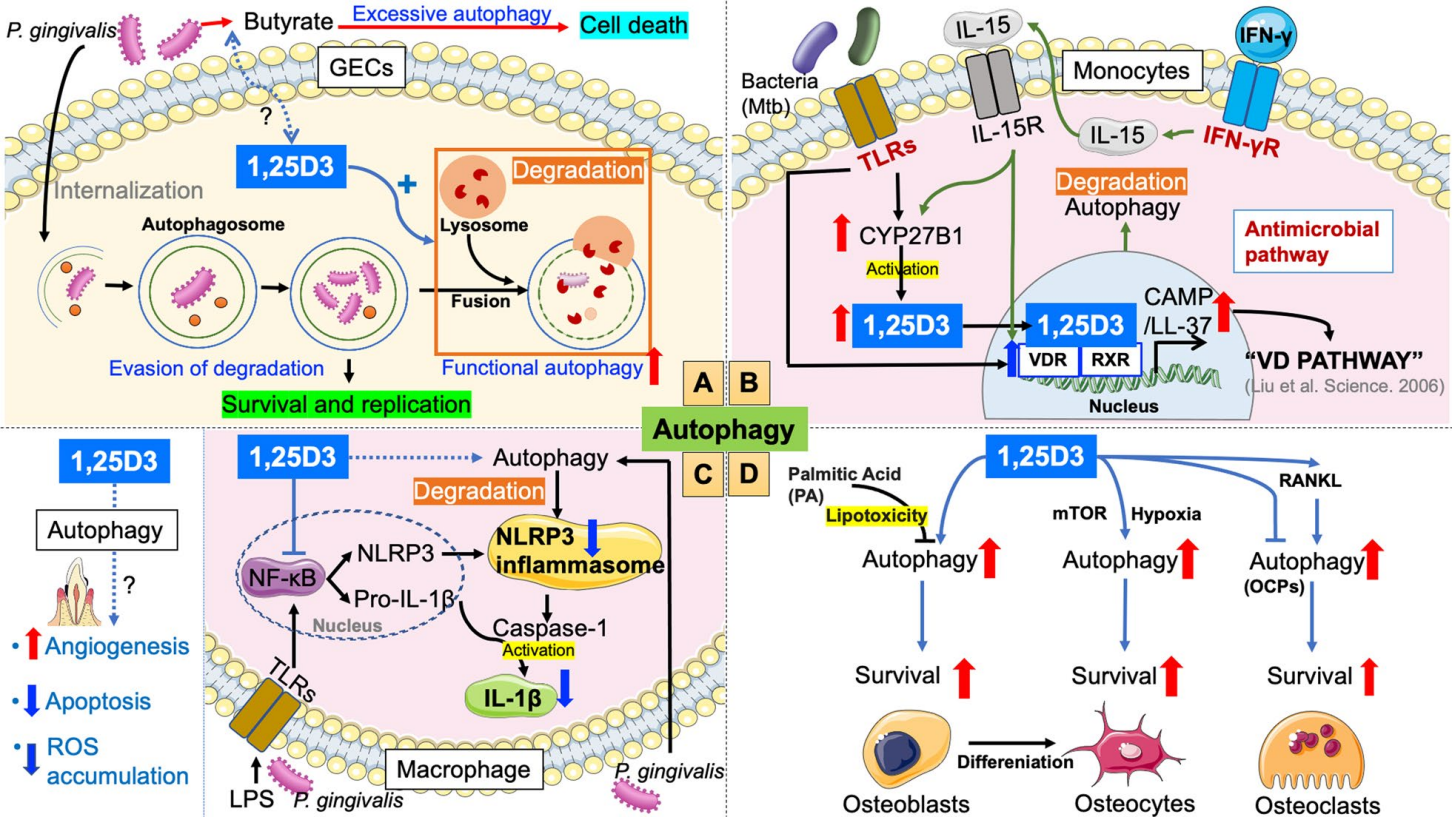
Possible role of 1,25D3 via autophagy modulation in the pathogenesis of periodontitis. **A** *P. gingivalis*-induced autophagy provides a favorable microenvironment for its replication and survival, whereas 1,25D3 could convert this impaired autophagy into a functional one by promoting fusion with lysosomes. Butyrate activates cell death via excessive autophagy in GECs and gingival fibroblasts. Whether there is an interaction between 1,25D3 and butyrate in periodontal tissue remains unknown. **B** TLRs activation by bacteria (such as Mtb) on monocytes upregulates the expression of VDR and 1-hydroxylase genes (CYP27B1), thereby leading to CAMP production and subsequent antimicrobial activity. The VD pathway was first described by Liu et al. in [91]. Similarly, 1,25D3-mediated autophagy was required for IFN-γ-induced antimicrobial activity. **C** 1,25D3 has been found to upregulate AhR expression, thus blocking NF-κB and NLRP3 which lead to tissue destruction, promote autophagy-mediated degradation of NLRP3 and downregulate IL-1β expression mediated by the NLRP3 inflammasome. Autophagy protects cells from apoptosis under inflammatory conditions, reduces ROS accumulation, and promotes angiogenesis in patients with periodontitis; however, whether 1,25D3 can induce autophagy in patients with periodontitis to exert such an effect is still unknown. **D** An increase in autophagy can promote the differentiation, survival, and normal functions of osteoblasts, osteoclasts, and osteocytes. 1,25D3 restores PA-mediated impaired autophagy to protect osteoblasts from lipotoxicity of PA and inhibits cell death of osteocytes in an mTOR pathway-dependent manner under hypoxic conditions. 1,25D3 plays a dual role in regulating the autophagy of OCPs, a process dependent on the RANKL intervention status; it inhibits autophagy of OCPs in the absence of RANKL and enhances RANKL-induced autophagy if the OCPs to exert a pro-osteoclastogenesis effect

Autophagy activated in infected cells is involved in intracellular antimicrobial defense mechanisms via a lysosomal degradation pathway [70]. Active 1,25D3 mediating autophagy enhances *Salmonella* clearance in intestinal epithelia and appears to be a promising treatment strategy for the control of Mtb infection [71]. In periodontal tissue, *P. gingivalis*, a major opportunistic pathogen, can induce autophagy with different functions in phagocytic (macrophages and dendritic cells) and non-phagocytic cells (GECs, endothelial cells, and gingival fibroblasts) after internalization [72–75]. Autophagy enhances the clearance of *P. gingivalis* internalized by macrophages and dendritic cells. However, to avoid clearance by the host immune system, *P. gingivalis* has developed specific survival strategies against GECs. In GECs and human coronary artery endothelial cells (HCAECs), *P. gingivalis* impairs the formation of autolysosomes to escape lysosomal degradation and replicate inside autophagosome vacuoles for persistent intracellular survival [70, 75]. *P. gingivalis*-induced autophagy provides a favorable microenvironment for replication, survival, and dissemination in the GECs and HCAECs, indicting its significant role in the progression of periodontitis and atherosclerosis [70, 76]. Interestingly, under active 1,25D3 treatment, the disabled autophagy induced by *P. gingivalis* in epithelial cells could become effective via increased number of autophagosome vacuoles and promoted fusion of autophagosomes and lysosomes. 1,25D3 significantly reduced the number of live *P. gingivalis* internalized into HeLa cell subline KB cells and U937 cells by promoting autophagy in a dose-dependent manner (Fig. 4A). The antibacterial effect of 1,25D3 greatly decreased after autophagy inhibition with 3-methyladenine (3-MA) treatment [65]. *A. actinomycetemcomitans* infection induced autophagy in human junctional epithelium keratinocytes (JEKs); this process inhibits the intracellular survival of the bacteria and significantly reduces the number of JEKs undergoing cell death [77]. 1,25D3 treatment enhances antibacterial activity to decrease the number of viable colonies of *A. actinomycetemcomitans* in cultured GEC [19]. However, whether its antibacterial mechanism is related to the regulation of autophagy and whether 1,25D3 plays a protective role against cell death via autophagy induction requires further exploration.

Interestingly, excessive autophagy or inadequate activation of autophagy may lead to cellular damage or even death [78]. Butyrate is a metabolite of some anaerobic periodontal bacteria that activate cell death via autophagy in the GECs and gingival fibroblasts. It is highly concentrated in the periodontal pocket and plays an important role in the initiation and progression of periodontal disease [79, 80]. However, butyrate also has a protective effect against infection in the gut. Butyrate produced by gut microbes upregulated VDR expression in a dose-dependent manner in human intestinal epithelial cells, and a decrease in the proliferation of butyrate-producing bacteria was observed in mice intestinal epithelia lacking VDR [81]. The reasons behind the different functions of butyrate at different sites remain unclear (Fig. 4A). Investigating the relationship between 1,25D3 and butyrate in the oral cavity may help us better understand the regulatory role of 1,25D3 in the progression of periodontal diseases.

### Immune regulation

1,25D3 plays a pivotal role in regulating immunity through autophagy, providing an antimicrobial defense mechanism against pathogens that invade immune cells. 1,25D3-induced autophagy is critical for the elimination of intracellular Mtb in human monocytes/macrophages [71], and cathelicidin is considered as an essential mediator of 1,25D3-induced autophagy [58]. Interestingly, the pathway through which IFN-γ promotes antimicrobial activity is dependent on 1,25D3 signaling-induced autophagy in human macrophages [82]. 1,25D3 is reported to induce autophagy in a cathelicidin-independent manner for the inhibition of human immunodeficiency virus type-1 (HIV-1) replication in macrophages [83]. 1,25D3 provides a therapeutic strategy for viral infections, such as the influenza virus, by restoring the autophagic flux, thereby preventing apoptosis [84]. In periodontal diseases, the induction of autophagy enhances the killing of periodontal pathogens that invade into the macrophages and dendritic cells. In THP-1-derived macrophages, the intracellular survival of *P. gingivalis* and *A. actinomycetemcomitans* is inhibited by enhanced autophagy [73, 85]. It has been reported that after 1,25D3 treatment, the amount of *P. gingivalis* in U937-derived macrophages decreased in a dose-dependent manner. Its mechanism of action may be related to the degradation of live *P. gingivalis* due to the 1,25D3-promoted co-localization of *P. gingivalis* with autophagosome and lysosomal markers [86]. Moreover, the survival of *P. gingivalis* within dendritic cells is impaired by rapamycin-induced autophagy [72]. The recognition of *P. gingivalis* by dendritic cells results in two scenarios: blocking autophagy for survival and promoting autophagy for degradation. Using autophagy promoters could help promote the killing of pathogens and periodontitis resolution, thus providing insights into a novel therapeutic approach [87].

In addition, autophagy has become more interrelated with TLRs signaling. TLR signaling stimulated by TLR ligands is important for the initiation and regulation of autophagy activation [88]. Additionally, 1,25D3/VDR signaling is involved in the TLR-induced autophagic pathway. 1,25D3-dependent autophagy is induced by TLR signaling. For example, TLR2/1/CD14 stimulation by mycobacterial lipoprotein LpqH increased the mRNA expression of Cyp27b1 hydroxylase and functional VDR activation in a time-dependent manner, thereby inducing autophagy in human monocytes [89]. The interaction between the 1,25D3/VDR-AMP axis and autophagy is currently a hot research topic [90]. In 2006, Liu et al. first named the reaction in monocytes caused by the activation of Toll-like receptors (TLRs) by bacteria during the production of CAMP as the VD pathway. TLR activation by bacteria on macrophages could upregulate the expression of VDR and 1-hydroxylase genes, thereby leading to CAMP production and subsequent antimicrobial activity [91]. This pathway also exists in HGECs, HGFs, and HPDLCs infected by *P. gingivalis* [32, 92, 93]. These results indicate the pathway in which TLRs induce 1,25D3-dependent antibacterial activity against intracellular bacteria. Insufficient 1,25D3 levels in the body may lead to a reduction in TLR-induced antibacterial activity, thereby increasing the risk of periodontitis (Fig. 4B).

Autophagy is also considered a regulator of T cells, affecting T cell function, differentiation, and metabolism [94]. In patients with active systemic lupus erythematosus, severe VD deficiency affects the expression of ATG proteins (mTOR and LC3) and leads to a significant increase in CD4^+^ T cell counts and a decrease in CD8^+^ T cell counts [54].

### Inflammation regulation

Autophagy activation can limit excessive inflammation in periodontal tissue by inhibiting IL-1β secretion, NLRP3 inflammasome formation, and reactive oxygen species (ROS) accumulation [73, 95–97], protecting cells from apoptosis under inflammatory conditions [63] and promoting angiogenesis [98–101] (Fig. 4C).

IL-1β amplifies periodontal inflammation and plays an important role in tissue destruction. LPS-induced p-p65 activates the NLRP3 inflammasome in immune cells by binding to NF-κB sites in the promoter region of NLRP3 [102]. The NLRP3 inflammasome, which is responsible for IL-1β secretion, significantly contributes to alveolar bone resorption by promoting osteoclast differentiation, and NLRP3 knockout reduced pathological alveolar bone loss in experimental periodontitis [103, 104]. As mentioned in SubSect. 1, 1,25D3 has been shown to inhibit NLRP3 and NLRP3-mediated IL-1β expression to attenuate experimental periodontitis in mice and reduce oral keratinocyte apoptosis. Little is known about whether autophagy mediates 1,25D3-induced anti-inflammatory and anti-apoptotic effects in periodontal disease. However, some connections have also been found in other diseases. In LPS-primed primary peritoneal macrophages in a mouse model, 1,25D3 has been found to promote autophagy-mediated degradation of NLRP3 and downregulate IL-1β expression mediated by the NLRP3 inflammasome [105] (Fig. 4C). ROS, an important element in NLRP3 activation, was found to be significantly decreased after 1,25D3 treatment in peritoneal macrophages [105]. 1,25D3 treatment increases autophagy in skin flaps, which might contribute to the reduction of oxidative stress, thereby significantly enhancing skin flap survival [106].

In addition, 1,25D3 is known to induce autophagy to inhibit apoptosis in some diseases. For instance, 1,25D3 prevents influenza virus-induced cellular apoptosis by restoring autophagic flux, providing a therapeutic strategy for viral infection [84] (Fig. 4C).

Since VDR exists widely in vascular endothelial cells and smooth muscle cells, the regulatory role of 1,25D3 in angiogenesis and vascular cell activity has been reported [107]. Studies have demonstrated the promotion of vascularization by 1,25D3 in skin flaps [106]. However, 1,25D3 was also reported to reduce retinal and corneal neovascularization in mice [108]. These results suggest that the role of 1,25D3 in the regulation of angiogenesis varies in different diseases. In addition, the pro-angiogenic ability of autophagy has been investigated in the periodontium. Autophagy promotes angiogenesis mediated by the mesenchymal stem, including PDLSCs [99, 100]. Activation of autophagy by rapamycin in PDLSCs was found to increase the secretion of angiogenesis-promoting cytokines such as angiogenin and basic fibroblast growth factor, whereas inhibition of autophagy with knockdown of Beclin1 led to the suppression of pro-angiogenic ability [101]. The above results provide new insights into the potential autophagy-mediated angiogenesis by 1,25D3 in the periodontium (Fig. 4C).

### Bone homeostasis

Alveolar bone homeostasis is tightly controlled by the balance between osteoclastogenesis and osteoblastogenesis. In periodontitis, an imbalance favoring bone resorption leads to pathological resorption of alveolar bone [109]. Autophagy, a new player, identified in recent years, plays an important role in bone homeostasis and is involved in the regulation of alveolar bone metabolism in the case of periodontitis [13, 110]. In general, autophagy is indispensable for the differentiation, survival, and normal functions of bone cells (including osteoclasts, osteoblasts, and osteocytes); thus, impaired autophagy could lead to bone pathologies [111–114]. For example, autophagy contributes not only to the survival of osteoblasts under oxidative stress [113, 114] and provides energy sources for osteoblast differentiation [115] but also to osteoclast reabsorption [114]. Autophagy is also involved in the terminal differentiation of osteoblasts into osteocytes and plays an important role in osteocyte survival [116]. During this process, autophagy adjusts the size and content of organelles and helps cells adapt to hypoxia and poor nutritional conditions and store energy, thus hindering bone loss [111]. Moreover, enhanced autophagy in osteoblasts has been shown to diminish bone resorption associated with inflammation, such as apical periodontitis [117].

The above findings suggest that the regulation of autophagy in bone cells may have therapeutic implications [110]. 1,25D3, a key autophagy regulator, promotes osteoblast production and protects osteoblasts from apoptosis [118, 119]. Autophagy may be an emerging mechanism through which 1,25D3 regulates bone cell differentiation and function (Fig. 4D). Recent studies have investigated the role of 1,25D3 in bone metabolism through the regulation of autophagy. For example, 1,25D3 protects osteoblasts from palmitate-induced lipotoxicity in vitro by regulating impaired autophagy to functional autophagy, thereby improving cell survival and function [119]. 1,25D3 plays a dual role in the autophagy of osteoclasts. In the absence of RANKL, 1,25D3 directly inhibits the autophagy of osteoclast precursors (OCPs). However, due to its positive impact on RANKL signaling, 1,25D3 could increase RANKL-induced autophagy of OCPs, eventually leading to a net pro-osteoclastogenesis effect. RANKL-induced osteoclastogenesis was dramatically decreased by the addition of autophagy inhibitors, further supporting the pro-osteoclastogenesis effect of 1,25D3 via autophagy [120]. 1,25D3 has also been found to inhibit osteocyte death under hypoxic conditions in an mTOR pathway-dependent manner. This raises the possibility of using 1,25D3 as a therapeutic intervention for conditions in which osteocyte death occurs under hypoxia [121]. Further, diabetes mellitus is known to be a major risk factor for periodontal disease, and these conditions are believed to be biologically associated with each other. Diabetes mellitus is associated with a high incidence of bone fractures and decreased bone density. 1,25D3 exerts an osteoprotective effect by reducing high-glucose-induced autophagy via the PI3K/Akt/FoxO1 signaling pathway, providing new insights on strategies for diabetes-induced bone loss [122].

## Conclusions

The protective role of 1,25D3 in the pathogenesis of periodontitis, including the clearance of periodontal pathogens, maintenance of the epithelial barrier, relief from inflammation, and reduction of alveolar bone loss, may be achieved, in part, through the regulation of autophagy. 1,25D3 signaling regulates autophagy, and that the regulation of autophagy is important for periodontal health. Given that autophagy is involved in the protective effect of 1,25D3 on infection, inflammation and bone metabolism in various diseases, further studies on the connection between 1,25D3 and autophagy in periodontitis may reveal the therapeutic potential of 1,25D3 and new strategies for periodontal prevention and treatment.

## Data Availability

Not applicable.

## Abbreviations

VDS: Vitamin D supplementation
VD: Vitamin D
1,25D3: 1,25-Dihydroxyvitamin D3
VDR: Vitamin D receptor
UVB: Ultraviolet B
7-DHC: 7-Dehydrocholesterol
VDBPs: Vitamin D-binding proteins
RXR: Retinoid X receptor
VDREs: Vitamin D response elements
MARRS: Membrane-associated, rapid response steroid-binding protein
AMPs: Antimicrobial peptides
CAMP: Cathelicidin antimicrobial peptides
TLRs: Toll-like receptors
HGECs: Human gingival epithelial cells
HGFs: Human gingival fibroblasts
HPDLCs: Human periodontal ligament cells
*P. gingivalis*: *Porphyromonas gingivalis*
*A. actinomycetemcomitans*: *Aggregatibacter actinomycetemcomitans*
LPS: Lipopolysaccharide
*F. nucleatum*: *Fusobacterium nucleatum*
*S. mutans*: *Streptococcus mutans*
ZO: Zonula occludens
ECJs: E-cadherin intercellular junctions
MMP-9: Matrix metalloproteinase-9
TNF-α: Tumor necrosis factor-α
NF-κB: Nuclear factor-κB
PUMA: P53-upregulated modulator of apoptosis
VHL: Von Hippel-Lindau
HIF-1α: Hypoxia-inducible factor-1α
IFNγ: Interferon γ
IL-: Interleukin-
MAPK: Mitogen-activated protein kinase
ERK-1/2: Extracellular signal-regulated kinase-1/2
AhR: Aryl hydrocarbon receptor
NLRP3: Nucleotide-binding oligomerization domain-like receptor family pyrin domain containing 3
RANKL: Receptor activator of NF-κB ligand
mTOR: Mammalian target of rapamycin
PI3KC3: Class III phosphoinositide 3-kinase
Mtb: *Mycobacterium tuberculosis*
ATG: Autophagy related
PTPN6: Protein tyrosine phosphatase non-receptor type 6
PBMCs: Peripheral blood mononuclear cells
HCAECs: Human coronary artery endothelial cells
3-MA: 3-Methyladenine
JEKs: Junctional epithelium keratinocytes
HIV-1: Human immunodeficiency virus type-1
ROS: Reactive oxygen species
ECs: Endothelial cells
AGEs: Advanced glycation end products
OCPs: Osteoclast precursors
